# Ubiquitination of the bacterial inositol phosphatase, SopB, regulates its biological activity at the plasma membrane

**DOI:** 10.1111/j.1462-5822.2009.01356.x

**Published:** 2009-08-04

**Authors:** Leigh A Knodler, Seth Winfree, Dan Drecktrah, Robin Ireland, Olivia Steele-Mortimer

**Affiliations:** Laboratory of Intracellular Parasites, Rocky Mountain LaboratoriesNIAID, NIH, Hamilton, MT 59840, USA

## Abstract

The *Salmonella* type III effector, SopB, is an inositol polyphosphate phosphatase that modulates host cell phospholipids at the plasma membrane and the nascent *Salmonella*-containing vacuole (SCV). Translocated SopB persists for many hours after infection and is ubiquitinated but the significance of this covalent modification has not been investigated. Here we identify by mass spectrometry six lysine residues of SopB that are mono-ubiquitinated. Substitution of these six lysine residues with arginine, SopB-K^6^R, almost completely eliminated SopB ubiquitination. We found that ubiquitination does not affect SopB stability or membrane association, or SopB-dependent events in SCV biogenesis. However, two spatially and temporally distinct events are dependent on ubiquitination, downregulation of SopB activity at the plasma membrane and prolonged retention of SopB on the SCV. Activation of the mammalian pro-survival kinase Akt/PKB, a downstream target of SopB, was intensified and prolonged after infection with the SopB-K^6^R mutant. At later times, fewer SCV were decorated with SopB-K^6^R compared with SopB. Instead SopB-K^6^R was present as discrete vesicles spread diffusely throughout the cell. Altogether, our data show that ubiquitination of SopB is not related to its intracellular stability but rather regulates its enzymatic activity at the plasma membrane and intracellular localization.

## Introduction

Post-translational modification by ubiquitin, or the ubiquitin-like (Ubl) proteins, is an important mechanism for regulating a broad range of biological processes. Ubiquitin is a small globular protein that is covalently attached to target proteins, most commonly at lysine residues. Ubiquitin itself contains seven lysine residues, allowing it to serve as a substrate for poly-ubiquitin chain formation. Similar to other post-translational modifications such as phosphorylation, ubiquitination is a dynamic, reversible process. The covalent attachment of ubiquitin to target proteins is catalysed in a three-step enzymatic cascade involving ubiquitin-activating (E1) and ubiquitin-conjugating (E2) enzymes and ubiquitin ligases (E3). The removal of ubiquitin occurs in one step, catalysed by deubiquitinases (Kerscher *et al*., 2006). Historically, the post-translational modification of proteins by ubiquitin has been associated with protein degradation (Mukhopadhyay and Riezman, 2007). Poly-ubiquitin chains of at least four Lys48-linked ubiquitin molecules efficiently target a protein for destruction by the 26S proteasome. More recently, non-proteolytic functions of ubiquitination have been revealed in many cellular pathways including membrane trafficking, DNA repair and replication and gene transcription (Welchman *et al*., 2005; Mukhopadhyay and Riezman, 2007; Chen and Sun, 2009). The Ubl proteins, a family of proteins related to ubiquitin, can also be covalently attached to lysine side-chains in target proteins and modify their function. Examples of Ubl proteins include interferon-stimulated gene-15 (ISG15), neural precursor cell-expressed developmentally downregulated-8 (NEDD8), small ubiquitin-related modifier (SUMO) and Atg8 (Welchman *et al*., 2005; Kerscher *et al*., 2006).

The ability of a pathogen to cause disease results from its being able to successfully compete at the host–pathogen interface. During the course of co-evolution, viral and bacterial pathogens have developed many strategies to manipulate host cells to their own advantage. Given the significance of ubiquitin and Ubl proteins to regulating mammalian cell processes, it is perhaps not surprising that many pathogens have targeted ubiquitin and Ubl conjugation pathways. For example, pathogens can regulate the host immune response by interfering with ubiquitin and Ubl protein-associated enzymes (Angot *et al*., 2007; Edelmann and Kessler, 2008). Alternatively, some pathogens encode virulence factors that are structural mimics of E3 ubiquitin ligases and deubiquitinases (Angot *et al*., 2007; Edelmann and Kessler, 2008). Viruses, protozoan and bacterial pathogens have all developed mechanisms to target these processes but this appears especially prevalent for Gram-negative bacteria that inject their own proteins, known as effectors, into host cells using type III (T3SS) or type IV secretion systems. The functions of virulence proteins can also be modified by ubiquitin or Ubl protein conjugation. For instance, the activity of some bacterial toxins and type III effectors is downregulated by poly-ubiquitin conjugation and proteasomal degradation, including listeriolysin O from *Listeria monocytogenes* (Schnupf *et al*., 2006), SopE and SopA from *Salmonella enterica* serovar Typhimurium (*S*. Typhimurium) (Kubori and Galan, 2003; Zhang *et al*., 2005), YopE from *Yersinia enterocolitica* (Ruckdeschel *et al*., 2006) and ExoT from *Pseudomonas aeruginosa* (Balachandran *et al*., 2007). Another two virulence proteins, SopB from *S*. Typhimurium (Marcus *et al*., 2001) and ExoU from *P. aeruginosa* (Stirling *et al*., 2006), are ubiquitinated, but this modification does not serve as a signal for proteasomal degradation. All of these examples exemplify two points; that the ubiquitin machinery is important to pathogenic virulence and the degree of cross-talk between pathogen and host at the level of ubiquitin and Ubl proteins is extensive.

The *Salmonella* type III effector, SopB, is an inositol phosphatase that acts on host cell membrane phospholipids. Its biological activity affects several facets of the interaction between *Salmonella* and mammalian cells. For example, its actions are required for optimal bacterial entry into non-phagocytic cells (Raffatellu *et al*., 2005), activation of the mammalian pro-survival kinase Akt/PKB (Steele-Mortimer *et al*., 2000) and maturation of the *Salmonella*-containing vacuole (SCV), by generating phosphatidylinositol (PI) 3-phosphate (PI(3)P) on the SCV and recruiting lysosomal glycoproteins to the SCV (Hernandez *et al*., 2004). It has previously been shown that SopB is membrane-associated and ubiquitinated after translocation into epithelial cells (Marcus *et al*., 2002). One unusual aspect of SopB activity is that it can be detected for several hours post invasion (Marcus *et al*., 2002; Drecktrah *et al*., 2005; Wasylnka *et al*., 2008), in contrast to most other ‘invasion-associated’*Salmonella* effector proteins that are active for only a short time during, and immediately following, the invasion process. For example, the ubiquitin-dependent proteolysis of SopE is crucial for the rapid downregulation of actin-based plasma membrane ruffling following internalization of *Salmonella* into epithelial cells (Kubori and Galan, 2003). The longevity of translocated SopB, in comparison, suggests that ubiquitination plays a different role in the regulation of this type III effector.

In order to clarify why SopB is post-translationally modified, we first determined which residues of SopB are ubiquitinated by mass spectrometry. We identified six lysine residues of SopB as ubiquitin conjugation sites. Specific mutation of these residues allowed us to test the function of ubiquitination and indicated that this post-translational modification regulates SopB activity without affecting its turnover.

## Results

### Translocation and ubiquitination of SopB continues for many hours post infection

**Fig. 1:**
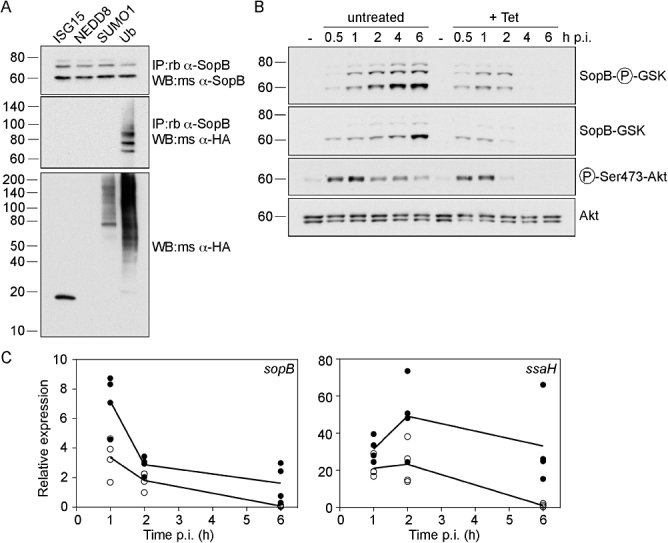
Translocated SopB is modified by ubiquitin, but not ubiquitin-like proteins. A. SopB is not modified by ISG15, NEDD8 or SUMO1. HeLa cells were transfected with HA-ISG15, HA-NEDD8, HA-SUMO1 or HA-ubiquitin (Ub) for 18 h prior to infection with Δ*sopB-sigE Salmonella* expressing plasmid-borne SopB and SigE under the control of the *sopB* promoter (pWSKDE). At 2 h p.i., cells were solubilized on ice for 15 min, then post-nuclear supernatants were incubated with rabbit polyclonal anti-SopB antibodies followed by Protein A Sepharose beads. Bound proteins were eluted with boiling 1.5× SDS-PAGE sample buffer, separated by SDS-PAGE and subject to immunoblotting with mouse polyclonal anti-SopB antibodies and mouse monoclonal anti-HA antibodies. Whole cell lysates were also probed with mouse anti-HA antibodies (bottom panel). Molecular mass markers in kDa are indicated on the left of each panel. B. Ubiquitinated SopB accumulates with time. HeLa cells were infected with Δ*sopB-sigE Salmonella* complemented with plasmid-borne SopB fused to a phosphorylatable tag derived from GSK, and its cognate type III chaperone, SigE (pWSKDE-GSK). At the indicated times, monolayers were solubilized, proteins were separated by SDS-PAGE and subject to immunoblotting with antibodies directed against phospho-Ser9-GSK 3β (to detect translocated SopB-GSK), GSK tag (total SopB-GSK), phospho-Ser473-Akt (activated Akt) and Akt pan (total Akt). Where indicated, 10 μg ml^−1^ tetracycline (+ Tet) was added at 30 min p.i. to stop bacterial protein synthesis. C. Taqman^®^ quantitative PCR analysis of *sopB* and *ssaH* transcript levels. HeLa cells were infected with wild-type *Salmonella* and samples were processed for quantitative PCR. To stop bacterial transcription after internalization, 5 μg ml^−1^ rifampicin was added at 10 min p.i. *sopB* and *ssaH* transcripts are normalized to *ftsZ* transcript amounts. Each symbol represents the mean from one experiment. The lines represent the statistical mean from four independent experiments. Closed circles, untreated. Open circles, rifampicin-treated cells.

Post-translational modification of eukaryotic proteins by ubiquitin or Ubl modifiers is an important process by which the stability, localization or activity of intracellular proteins can be controlled (Kerscher *et al*., 2006). Recently, ubiquitination of several pathogenic proteins, including the *Salmonella* effector protein SopB (Marcus *et al*., 2002), has been demonstrated. To investigate this process in more detail, we first asked whether SopB is also a substrate for conjugation by the Ubl modifiers ISG15, NEDD8 or SUMO1. HeLa cells expressing haemagglutinin (HA)-tagged forms of the modifiers were infected with *Salmonella* for 2 h, to allow for maximal translocation of SopB. Then total SopB, incorporating the intrabacterial pool as well as translocated protein, was immunoprecipitated from lysates and analysed by immunoblotting. Whole cell lysates were probed with anti-HA antibodies to detect ectopic expression levels. HA-SUMO and HA-ubiquitin were incorporated into multiple high-molecular-weight proteins, whereas HA-ISG15 and HA-NEDD8 monomers were primarily detected, at 18 and 6 kDa respectively (Fig. 1A and not shown). This is in agreement with the known prevalence of SUMO and ubiquitin substrates compared with ISG15 and NEDD8 (Kamitani *et al*., 1997; Welchman *et al*., 2005). When the immunoprecipitates were probed with an anti-SopB antibody, three SopB bands were detected in all of the lysates, migrating at ∼60, ∼68 and ∼75 kDa (Fig. 1A). By contrast, anti-HA antibodies only detected proteins in co-immunoprecipitates from cells expressing HA-ubiquitin, indicating that SopB is not modified by ISG15, NEDD8 or SUMO1. In the HA-ubiquitin sample, two distinct bands at ∼68 kDa and ∼75 kDa, and a slower migrating smear > 85 kDa were clearly resolved. These presumably represent mono-, di- and poly-ubiquitinated species of SopB respectively. We conclude from these immunoprecipitation experiments that ubiquitin, but not Ubl modifiers, targets SopB in infected cells.

We next followed a time-course of SopB ubiquitination during *Salmonella* infection of epithelial cells. In order to differentiate between the two intracellular pools of SopB; (i) translocated and (ii) intrabacterial, we used a recently described methodology based upon a 13-amino-acid tag derived from glycogen synthase kinase 3β (GSK) (Garcia *et al*., 2006). Because this tag is phosphorylated by serine protein kinases in mammalian cells, but not in bacteria, it can be used in conjunction with phospho-specific antibodies as a reporter for translocation. For these experiments bacteria lacking both SopB and its chaperone SigE (Δ*sopB-sigE*) were complemented with a low copy number plasmid encoding SopB-GSK and SigE (pWSKDE-GSK), expressed under the control of the *sopB* transcriptional promoter. Lysates prepared from infected HeLa cells at 0.5–6 h post infection (p.i.) were subject to immunoblot analysis using phospho-specific anti-GSK antibodies to detect translocated SopB-GSK. Three bands were revealed; one migrating at the predicted molecular mass of unmodified SopB (60 kDa), and two slower migrating species representing mono- and di-ubiquitinated SopB (Fig. 1B). While all three bands accumulated with time, indicating that both translocation and ubiquitination of SopB continued for several hours following infection, amounts of unmodified SopB always exceeded the ubiquitinated forms. It is important to note that SopB is translocated to the plasma membrane upon initial contact with host cells and after bacterial internalization to the SCV, therefore this profile represents a mixture of translocated SopB in these two spatially separate locales. Antibodies that recognize total GSK showed a similar increase in SopB-GSK with time, although the proportion of unmodified SopB (60 kDa) was more prominent than for the translocated SopB profile, suggesting that a significant proportion of SopB remains intrabacterial. To ensure that the GSK-tagged SopB was biologically active, we assessed the levels of phosphorylated Akt (phospho-Ser473 antibodies) in the lysates because SopB induces the phosphorylation of this eukaryotic kinase in epithelial cells (Steele-Mortimer *et al*., 2000; Knodler *et al*., 2005). Akt phosphorylation kinetics were similar to that shown previously, with a peak at 0.5–1 h p.i. (Knodler *et al*., 2005), but interestingly this was not coincident with maximal levels of translocated SopB (≥ 4 h p.i.).

The prolonged accumulation of translocated SopB is unexpected for a type III effector that is part of the *Salmonella* pathogenicity island 1 regulon. We used real-time PCR to study the levels of *sopB* transcription after bacterial internalization. HeLa cells were infected with wild-type bacteria and samples were processed for quantitative real-time PCR analysis at various times p.i. To prevent bacterial transcription after internalization, rifampicin was added at 10 min p.i. Transcript levels of *sopB* and *ssaH*, a gene encoding a structural component of T3SS2, were compared in the absence and presence of rifampicin over the time-course (Fig. 1C). Similar to phagocytic cells, the expression of *sopB* rapidly decreased after bacterial internalization (Drecktrah *et al*., 2006). However, *sopB* transcript levels were higher in untreated cells than for rifampicin-treated cells up to 6 h p.i. By comparison, the T3SS2 apparatus gene *ssaH* was induced intracellularly, in agreement with previous data from phagocytic cells (Cirillo *et al*., 1998; Drecktrah *et al*., 2006). Rifampicin treatment effectively diminished this intracellular induction, confirming that bacterial transcription was effectively prevented. From these data we conclude that, despite a net downregulation of *sopB* after internalization, bacteria continue to transcribe this gene up to 6 h p.i. Next we examined whether the accumulation of translocated SopB with time also requires protein synthesis after internalization. HeLa cells were infected with Δ*sopB-sigE* bacteria complemented with pWSKDE-GSK and treated with tetracycline at 30 min p.i. to block bacterial protein synthesis. Whole cell lysates were then subject to immunoblot analysis for phospho-GSK (translocated SopB) and GSK tag (total SopB) (Fig. 1B). Tetracycline treatment effectively prevented the accumulation of all forms of SopB, demonstrating that *de novo* protein synthesis is required for the prolonged accumulation of SopB in host cells. We conclude from these experiments that *sopB* continues to be transcribed and translated for many hours after bacterial internalization.

### Six lysine residues of SopB serve as ubiquitin acceptor sites

**Fig. 2:**
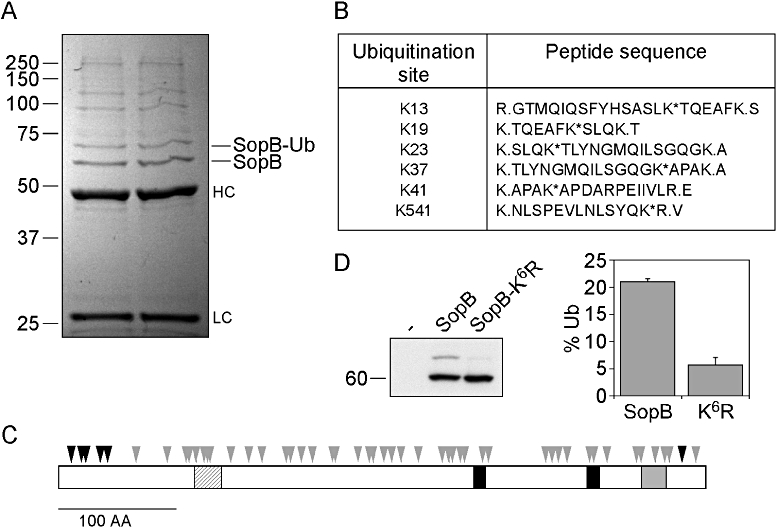
Identification of six lysine residues as ubiquitin conjugation sites. A. Immunopurification of SopB. HeLa cells ectopically expressing Myc-SopB were lysed and post-nuclear supernatants were incubated with anti-Myc monoclonal antibodies followed by Protein A Sepharose. Bound proteins were separated by SDS-PAGE and visualized using GelCode Blue reagent. Bands corresponding to unmodified SopB (SopB) and ubiquitinated SopB (SopB-Ub) were excised and subject to mass spectrometric analysis. HC, heavy chain; LC, light chain. B. Six ubiquitin-conjugated tryptic peptides were identified by mass spectrometry. Asterisks indicate the ubiquitinated lysine residues. Periods indicate the sites of trypsin cleavage. C. Schematic of SopB. Arrowheads indicate the 50 lysine residues present in SopB. Black arrowheads denote the six lysines that were identified as ubiquitin conjugation sites. Black boxes and a grey box represent regions with homology to mammalian 4′-phosphatases and 5′-phosphatases respectively. The hatched box depicts a predicted coiled-coil domain. D. Mutation of the six identified lysine residues impairs ubiquitination of transfected SopB. Left panel: HeLa cells were untreated (–) or transiently transfected for 18 h with Myc-SopB (SopB) or Myc-SopB K13R K19R K23R K37R K41R K541R (SopB-K^6^R). Monolayers were lysed in boiling 1.5× SDS-PAGE sample buffer, proteins separated by SDS-PAGE and subject to immunoblotting with mouse polyclonal anti-SopB antibodies. Right panel: Densitometric analysis of unmodified and monoubiquitinated SopB bands was used to determine the amount of SopB that was modified. Results are means ± SD from three separate experiments. *P* < 0.001, Student's *t*-test.

To further characterize the ubiquitination of SopB we used mass spectrometry analysis to identify conjugated lysine residues. As SopB contains 50 lysine residues that are potential ubiquitin conjugation sites, mutational analysis would not be practical. As an alternative, HeLa cells were transfected with a plasmid encoding Myc-tagged SopB (pCMV-Myc-SopB) and the protein purified by immunoprecipitation using an anti-Myc antibody, in order to obtain sufficient material for analysis. We have previously shown that, like translocated SopB, ectopically expressed SopB is ubiquitinated in mammalian cells (Marcus *et al*., 2002). Immunopurified SopB was separated by SDS-PAGE, visualized by GelCode Blue staining (Fig. 2A) and the regions containing unmodified SopB and ubiquitinated SopB (SopB-Ub) were excised, digested with trypsin and analysed by capillary liquid chromatography tandem mass spectrometry. Trypsin digestion of ubiquitinated proteins produces unique signature peptides which can be identified by their additional mass of 114.043 Da, corresponding to the –GG remnant from ubiquitin that remains conjugated to lysine residues (Xu and Peng, 2006). The Ubl proteins, NEDD8 and ISG15, also produce the same –GG signature tag when cleaved by trypsin. Modification by ubiquitin and Ubl proteins also causes a missed trypsin cleavage at the modified lysine residue (Xu and Peng, 2006). As we had already shown that SopB is not modified by either NEDD8 or ISG15 (Fig. 1A), any lysine modification would be due to ubiquitin conjugation only. Mass spectrometry analysis revealed no –GG signature peptides in the sample containing unmodified SopB, with peptide coverage of ∼80% of the SopB sequence. By contrast, six ubiquitination sites were identified for SopB-Ub; Lys13, Lys19, Lys23, Lys37, Lys41 and Lys541 (Fig. 2B), with detected peptides covering ∼79% of the SopB sequence. The identification of multiple modification sites, together with a single SopB-Ub band on SDS-PAGE gels, suggests that SopB is mono-ubiquitinated on any one of six lysine residues at once, but not all six residues simultaneously. Interestingly, five of the identified lysines are clustered at the N-terminus of SopB (Fig. 2C, black arrowheads).

In order to characterize the requirement for these six lysines in SopB ubiquitination, site-directed mutagenesis was used to create lysine-to-arginine substitutions in the pCMV-Myc-SopB plasmid. Following ectopic expression in transfected HeLa cells, the level of post-translational modification was monitored by immunoblotting of whole cell lysates with anti-SopB antibodies. Ectopically expressed SopB appeared as two bands in immunoblots, representing unmodified and mono-ubiquitinated SopB (Fig. 2D) (Marcus *et al*., 2002). Single lysine-to-arginine substitutions had no detectable effect on the ubiquitination status of SopB (results not shown), indicating that no single lysine residue is essential or preferred as a conjugation site. However, ubiquitination of a SopB mutant in which all six lysine residues were substituted (SopB-K^6^R) was substantially decreased (Fig. 2D). The residual ubiquitination presumably occurs on one or more additional residues that were not identified by mass spectrometry.

**Fig. 3:**
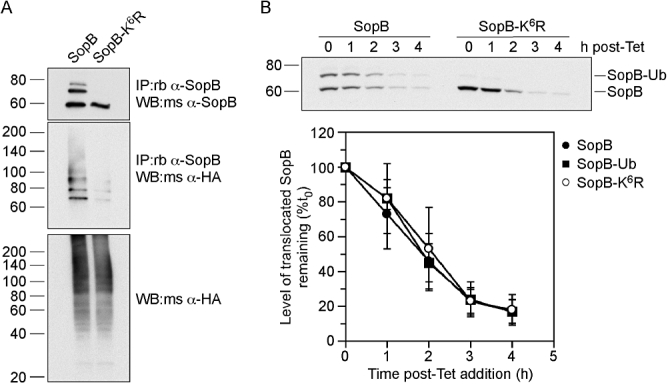
Ubiquitination of SopB does not alter its stability. A. Translocated SopB-K^6^R is defective for ubiquitination. HeLa cells expressing HA-ubiquitin were infected with Δ*sopB-sigE Salmonella* expressing plasmid-borne SopB (SopB) and SigE, or SopB with six lysine mutations (SopB-K^6^R) and SigE. At 2 h p.i., cells were solubilized and post-nuclear supernatants incubated with rabbit polyclonal anti-SopB antibodies followed by Protein A Sepharose beads. Bound proteins were eluted, separated by SDS-PAGE and subject to immunoblotting with mouse polyclonal anti-SopB antibodies or mouse monoclonal anti-HA antibodies. Whole cell lysates were also probed with anti-HA antibodies. B. Ubiquitination does not affect the stability of translocated SopB. HeLa cells were infected with Δ*sopB-sigE Salmonella* harbouring pWSKDE-GSK or pWSKDE-K^6^R-GSK. At 2 h p.i., 10 μg ml^−1^ tetracycline was added to the infected monolayers to stop bacterial protein synthesis and whole cell lysates were collected at hourly intervals by adding boiling 1.5× SDS-PAGE sample buffer. The stability of translocated SopB-GSK was monitored by immunoblotting with rabbit polyclonal antibodies against phospho-GSK. Graph shows densitometric analysis from three separate experiments (mean ± SD).

Having shown that ectopically expressed SopB can be mono-ubiquitinated on at least six different lysine residues, we next assessed whether this is also true after bacterial translocation of SopB. Site-directed mutagenesis was used to create lysine-to-arginine substitutions at all six residues in plasmid borne SopB. The resultant plasmid, pWSKDE-K^6^R, was then used to complement the Δ*sopB-sigE* mutant. HeLa cells expressing HA-ubiquitin were infected with either Δ*sopB-sigE* pWSKDE or Δ*sopB-sigE* pWSKDE-K^6^R bacteria. At 2 h p.i., monolayers were lysed and subject to immunoprecipitation with anti-SopB antibodies. Immunoprecipitates were subsequently analysed by immunoblotting for SopB and HA (Fig. 3A). Immunoprecipitated SopB migrated as three distinct bands on SDS-PAGE, representing the unmodified, mono- and di-ubiquitinated forms of SopB. By contrast, SopB-K^6^R migrated as a single species at 60 kDa, the same molecular mass as unmodified SopB. Furthermore, co-immunoprecipitation of HA-ubiquitin was almost completely eliminated for SopB-K^6^R, compared with wild-type SopB. Collectively, these data demonstrate that multiple lysines in SopB can be mono-ubiquitinated, and mutation of all these lysine residues (K13R, K19R, K23R, K37R, K41R, K541R) impairs ubiquitination of translocated SopB.

### Ubiquitination does not affect SopB stability or membrane association

We next evaluated the role of SopB ubiquitination on protein stability and localization, two known functions of this post-translational modification. To monitor the stability of translocated SopB, a modified pulse-chase experiment was used. HeLa cells were infected with Δ*sopB-sigE* bacteria complemented with either pWSKDE-GSK or pWSKDE-K^6^R-GSK. At 2 h p.i., tetracycline was added to stop bacterial protein synthesis. Samples were then taken at hourly intervals and whole cell lysates subject to immunoblotting with phospho-GSK antibodies to detect translocated SopB (Fig. 3B). After the addition of antibiotic, there was a steady decline in the amount of SopB, ubiquitinated SopB and SopB-K^6^R with time. Densitometric analysis showed that all three species had a half-life of approximately 2 h after translocation into the host cell. We conclude from these results that ubiquitination does not influence the degradation rate of SopB.

**Fig. 4:**
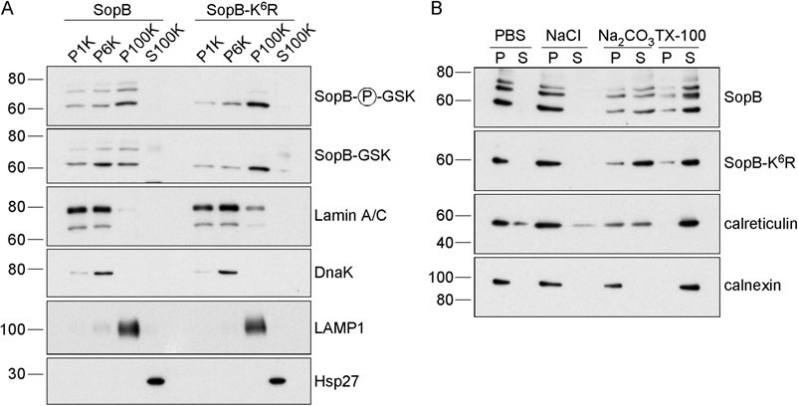
Membrane association of SopB is independent of ubiquitination. A. Translocated SopB and SopB-K^6^R fractionate to host cell membranes. HeLa cells were infected with *Salmonella* as described for Fig. 3A. At 1.5 h p.i., cells were mechanically fractionated and subject to differential centrifugation to obtain four fractions – P1K, P6K, P100K and S100K. Equal volumes were separated by SDS-PAGE and subject to immunoblotting with antibodies directed against phospho-GSK (translocated SopB), GSK tag (total SopB), lamin A/C (nucleus), DnaK (intact bacteria), LAMP1 (HeLa membranes) and Hsp27 (HeLa cytosol). B. Membrane association of translocated SopB and SopB-K^6^R is via hydrophobic interactions. HeLa cells were infected with *Salmonella* as described in Fig. 3A. At 1.5 h p.i., cells were mechanically fractionated and host cell membranes purified by ultracentrifugation. Membranes were extracted on ice with phosphate-buffered saline (PBS), high salt (1 M NaCl), sodium carbonate pH 11.5 (Na_2_CO_3_) or non-ionic detergent (TX-100). Insoluble (P) and soluble (S) fractions were then separated by ultracentrifugation. Equal volumes were subject to immunoblotting with antibodies against phospho-GSK (translocated SopB), calreticulin (peripheral membrane protein) or calnexin (integral membrane protein).

We have previously shown that SopB is targeted to host cell membranes after bacterial translocation (Marcus *et al*., 2002). We therefore considered whether ubiquitin affects the intracellular targeting of SopB. Epithelial cells were infected with Δ*sopB-sigE* bacteria complemented with either pWSKDE-GSK or pWSKDE-K^6^R-GSK and at 1.5 h p.i. cells were homogenized and subject to differential centrifugation at 1000 *g*, 6000 *g* and 100 000 *g* resulting in four fractions; P1K (nuclei), P6K (bacteria and nuclei), P100K (HeLa cell membranes), S100K (HeLa cell cytosol). The content of these fractions was assessed by immunoblotting with antibodies to the following proteins: lamin A/C, lysosomal-associated membrane protein 1 (LAMP1), Hsp27, DnaK, phospho-GSK (translocated SopB-GSK) and GSK-tag (total SopB-GSK) (Fig. 4A). The nuclear marker, lamin A/C, was equally detected in P1K and P6K fractions whereas DnaK, a marker for intact bacteria, was enriched in the P6K fraction, with only a small amount detected in P1K. No bacterial contamination (DnaK) was detected in the P100K or S100K fractions, which contained the host cell membrane and cytosolic proteins, LAMP1 and Hsp27 respectively. The three major forms of translocated SopB (60, 68 and 75 kDa) were primarily detected in the P100K fraction. The 60 kDa unmodified SopB and the 68 kDa mono-ubiquitinated SopB were also detected to a lesser extent in the P1K and P6K fractions. Importantly, all forms of SopB, including SopB-K^6^R, partitioned similarly. From these subcellular fractionation experiments we conclude that translocated SopB is enriched in host cell membrane fractions independent of its ubiquitination status.

We next asked whether ubiquitination of SopB could change the nature of its association with host cell membranes. Host cell membrane fractions were prepared from infected HeLa cells at 1.5 h p.i. as described above and then treated with either high salt, sodium carbonate or Triton X-100 (TX-100) on ice for 30 min. Subsequently, the samples were ultracentrifuged and equivalent volumes of soluble and insoluble fractions analysed by immunoblotting with antibodies against calnexin, calreticulin and phospho-GSK (Fig. 4B). Treatment of membranes with high salt or sodium carbonate pH 11.5 releases peripheral membrane proteins that are associated by ionic or hydrophobic interactions respectively, whereas integral membrane proteins are only solubilized by detergents (Helenius and Simons, 1975; Fujiki *et al*., 1982). Confirming this, the peripheral membrane protein, calreticulin, was extracted from membranes with sodium carbonate and TX-100, whereas the integral membrane protein, calnexin, was solubilized by detergent treatment only. Translocated SopB was removed from membranes with sodium carbonate and TX-100 treatments, but not high salt, comparable to what has previously been shown for ectopically expressed SopB (Marcus *et al*., 2002). There was no reproducible difference in the degree of solubilization of SopB, ubiquitinated SopB or SopB-K^6^R with the four treatments. Therefore, translocated SopB is peripherally associated with membranes by hydrophobic interactions, regardless of its ubiquitination.

### The association of SopB with the SCV is influenced by its ubiquitination

**Fig. 5:**
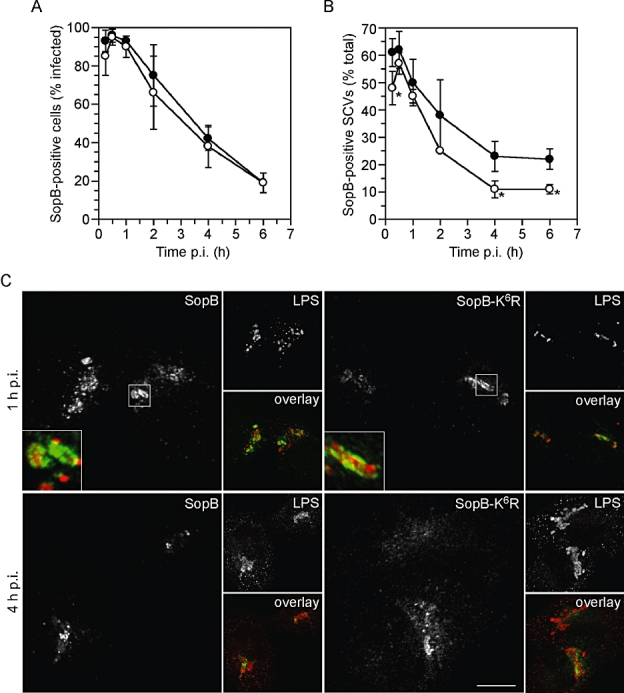
Ubiquitination affects the SCV association of SopB. A. Ubiquitination does not affect the incidence of SopB translocation. HeLa cells were infected with Δ*sopB-sigE* bacteria complemented with plasmid-borne SopB-2HA and SigE (pWSKDE-2HA), or SopB-K^6^R-2HA and SigE (pWSKDE-K^6^R-2HA). At various times p.i., monolayers were fixed and immunostained with antibodies against the HA-epitope tag and *Salmonella* LPS. The number of infected cells that were positive for SopB-2HA or SopB-K^6^R-2HA staining was scored visually by fluorescence microscopy. Mean ± SD (*n* > 150 infected cells per time point). Closed circles, SopB-2HA; open circles, SopB-K^6^R-2HA. B. Ubiquitination increases the association of SopB with the SCV. HeLa cells were infected and immunostained as for (A). For SopB-positive infected cells, the number of bacteria that were surrounded by SopB-2HA or SopB-K^6^R-2HA was scored. Mean ± SD from three separate experiments (> 300 bacteria per time point). Closed circles, SopB-2HA; open circles, SopB-K^6^R-2HA. Asterisks denote significantly different from wild-type SopB (*P*< 0.05, Student's *t*-test). C. Representative confocal microscopy images of translocated SopB-2HA and SopB-K^6^R-2HA at 1 h and 4 h p.i. At 1 h p.i., both SopB and SopB-K^6^R show ‘patchy’ staining around the bacteria, on the SCV. By 4 h p.i., there is less association of SopB and SopB-K^6^R with the SCV and SopB-K^6^R is primarily seen as punctate structures spread diffusely throughout the cell. Inset shows three times enlargement of indicated area. Scale bar is 10 μM.

One possibility is that ubiquitin-defective SopB remains associated with host cell membranes but has an altered intracellular location, so we compared the intracellular localization of SopB and SopB-K^6^R after translocation. HeLa cells were infected with Δ*sopB-sigE* bacteria complemented with HA-tagged SopB or SopB-K^6^R. At various times p.i., cells were fixed and immunostained with anti-HA antibodies to detect translocated SopB and anti-*Salmonella* lipopolysaccharide (LPS) antibodies. At early times p.i., most of the infected cells were positive for translocated SopB-2HA or SopB-K^6^R-2HA. With time, we observed an overall decrease in the number of infected cells positive for translocated SopB and SopB-K^6^R (Fig. 5A). Translocated SopB and SopB-K^6^R was detected as early as 15 min p.i., present in a patchy distribution on the SCV surrounding the bacteria (Fig. 5C), similar to what we have previously described (Marcus *et al*., 2002). Comparatively fewer SopB-K^6^R bacteria were positive for SCV staining at 15 min p.i. and the peak of SCV association was at 30 min p.i. for both SopB and SopB-K^6^R (Fig. 5B). While the immunostaining pattern for SopB and SopB-K^6^R was indistinguishable at early times p.i. (≤ 2 h p.i.), at later times, SopB-2HA remained associated with the SCV and was also detected in small vesicles in the vicinity of the SCV, whereas SopB-K^6^R-2HA was primarily detected as small punctate structures spread throughout the cell, appearing to have diffused away from SCVs (Fig. 5C). As a result, there was a significant difference in the number of SCVs decorated with SopB-2HA and SopB-K^6^R-2HA at 4 and 6 h p.i. (Fig. 5B). From these observations, we conclude that ubiquitination of SopB leads to its prolonged retention on the SCV.

### SopB-dependent SCV trafficking events are independent of its ubiquitination

**Fig. 6:**
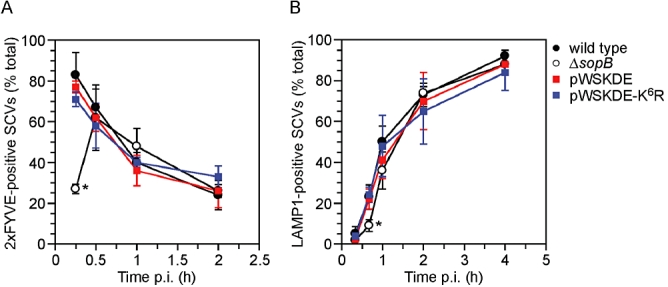
Ubiquitination of SopB is not required for early SCV trafficking events. A. HeLa cells expressing 2xFYVE-EGFP were infected with wild-type, Δ*sopB-sigE*, Δ*sopB-sigE* complemented with plasmid-borne SopB and SigE (pWSKDE) or plasmid-borne SopB-K^6^R mutant and SigE (pWSKDE-K^6^R). At the indicated times, monolayers were fixed and immunostained with anti-*Salmonella* LPS antibodies to differentiate intracellular and extracellular bacteria. The number of intracellular bacteria positive for 2xFYVE-EGFP, a probe for PI(3)P, was scored visually by fluorescence microscopy. Means ± SD from three separate experiments are shown (*n* ≥ 145 bacteria for each condition). Asterisk denotes significantly different from wild-type bacteria (*P*< 0.001, analysis of variance (anova) with Dunnett's *post hoc* test). B. HeLa cells were infected with *Salmonella* as described for (A) and at the indicated times monolayers were fixed and immunostained with rabbit polyclonal anti-*Salmonella* LPS antibodies and mouse monoclonal anti-human LAMP1 antibodies. The number of *Salmonella* positive for LAMP1 staining was scored visually by fluorescence microscopy. Means ± SD from three separate experiments are shown (*n* > 300 bacteria for each condition). Asterisk denotes significantly different from wild-type bacteria (*P*< 0.02, anova with Dunnett's *post hoc* test).

The biological activity of SopB contributes to several aspects of vacuole trafficking, including SCV maturation and positioning (Hernandez *et al*., 2004; Mallo *et al*., 2008; Wasylnka *et al*., 2008). To investigate if the ubiquitination of SopB plays a role in SCV biogenesis, we studied generation of the phospholipid, PI(3)P, and recruitment of the lysosomal glycoprotein, LAMP1, two processes that are affected by SopB. PI(3)P accumulates on the outer leaflet of SCVs immediately after bacterial entry, whereas LAMP1 is more gradually recruited to the SCV (Steele-Mortimer *et al*., 1999; Pattni *et al*., 2001; Hernandez *et al*., 2004). The double FYVE finger from Hrs was used as a specific probe for PI(3)P (Pattni *et al*., 2001). HeLa cells expressing a 2xFYVE-enhanced green fluorescent protein (EGFP) fusion were infected with *Salmonella* and at various times p.i. monolayers were fixed and immunostained with anti-*Salmonella* LPS antibodies to discriminate intracellular bacteria (Fig. 6A). For wild-type *Salmonella*, the peak of PI(3)P accumulation on SCVs was at 15 min p.i., and thereafter steadily declined. Markedly fewer SCVs containing Δ*sopB-sigE* bacteria were positive for the 2xFYVE probe at 15 min p.i., in agreement with previous reports (Mallo *et al*., 2008), but there was no significant difference in PI(3)P labelling of wild type and Δ*sopB* vacuoles at times ≥ 30 min p.i. Interestingly, a similar profile of early SopB-dependent, and later SopB-independent, Rab5 recruitment to the SCV has been reported (Mallo *et al*., 2008). Complementation of the Δ*sopB-sigE* mutant with wild-type SopB or SopB-K^6^R gave indistinguishable results from wild-type bacteria, demonstrating that SopB ubiquitination does not influence PI(3)P generation on the SCV.

A *sopB* mutant is delayed for SCV maturation, as assessed by the recruitment of lysosomal membrane glycoproteins such as LAMP1 (Hernandez *et al*., 2004). To determine if SopB ubiquitination affected this aspect of SCV trafficking, HeLa cells were infected with *Salmonella* and at various times p.i., monolayers were fixed and immunostained with antibodies against *Salmonella* LPS and LAMP1. We also found that SopB is required for efficient LAMP1 acquisition, but only at times < 1 h p.i. (Fig. 6B). Our complementation data showed that the ubiquitination of SopB does not contribute to vacuole maturation, because there was no difference in the kinetics of LAMP1 recruitment for SCVs containing bacteria expressing wild-type SopB or SopB-K^6^R. We conclude that the early SopB-dependent SCV trafficking events (< 1 h p.i.) are not influenced by the post-translational modification of SopB. This is consistent with the time-course of SopB ubiquitination, which is not evident until ≥ 1 h p.i. (Fig. 1B).

### Ubiquitination affects SopB activity at the plasma membrane

**Fig. 7:**
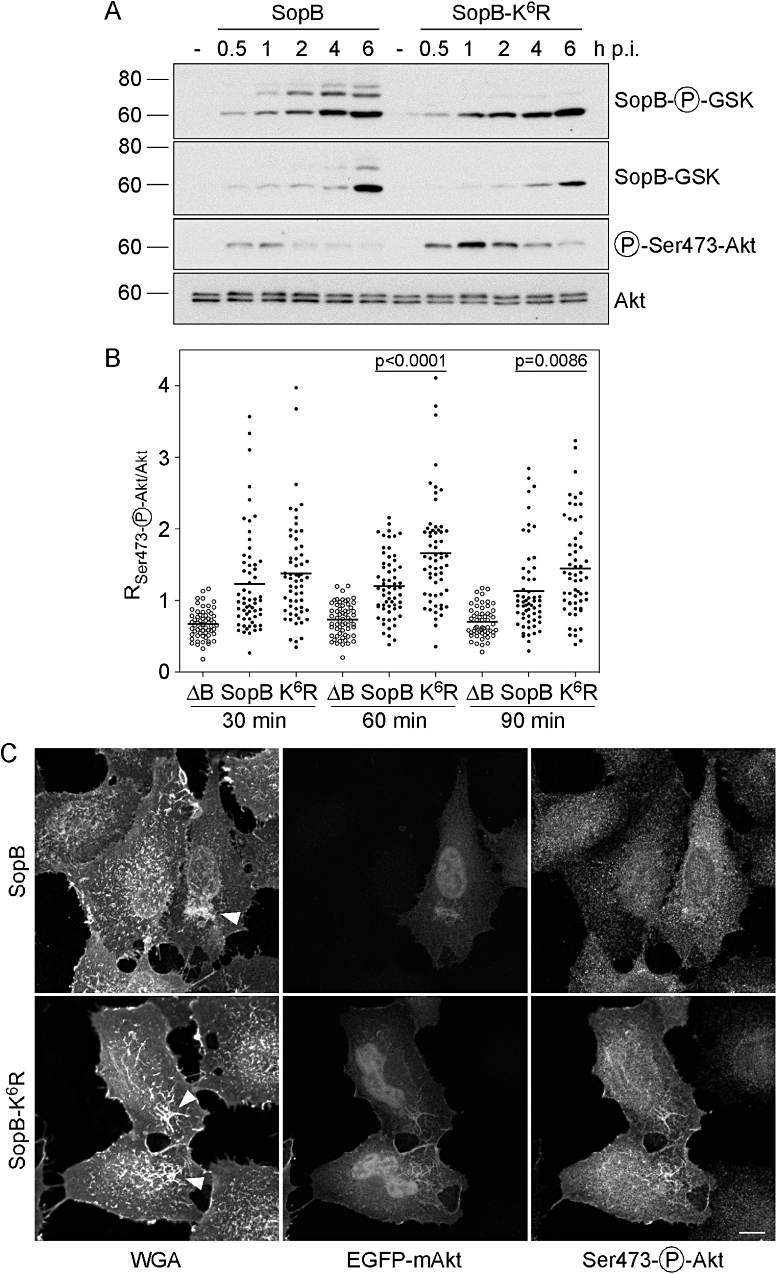
Ubiquitination of SopB downmodulates its activity at the plasma membrane. A. HeLa cells were infected with Δ*sopB-sigE Salmonella* complemented with either pWSKDE-GSK or pWSKDE-K^6^R-GSK. At the indicated times, monolayers were solubilized and proteins subject to immunoblotting with antibodies against phospho-GSK (translocated SopB), GSK tag (total SopB), phospho-Ser473-Akt (activated Akt) and Akt pan (total Akt). B. HeLa cells expressing EGFP-mAkt were infected with Δ*sopB-sigE Salmonella* (Δ*B*), or Δ*sopB-sigE Salmonella* complemented with pWSKDE (SopB) or pWSKDE-K^6^R (K^6^R). Monolayers were fixed, incubated with Alexa Fluor^®^647-conjugated WGA to label the plasma membrane, permeabilized and immunostained with anti-phospho-Ser473-Akt antibodies. Single optical sections through individual ruffles were acquired with a spinning disk confocal microscope. Regions of interest were selected by thresholding on the WGA image and transferred to the EGFP-mAkt and phospho-Ser473-Akt images. The ratio of mean fluorescence intensity for phospho-Akt : total Akt was subsequently determined. Each data point represents one ruffle. Data with means from three independent experiments are shown (*n* ≥ 55 ruffles for each condition). *P*-values were determined by Student's *t*-test. C. Representative confocal microscopy images from (B) at 60 min p.i. Arrowheads indicate *Salmonella*-induced membrane ruffles. Scale bar is 10 μM.

We have previously described the *Salmonella*-induced activation of the mammalian pro-survival kinase, Akt/PKB (Steele-Mortimer *et al*., 2000). Unlike SCV trafficking events, phosphorylation of Akt in infected cells is absolutely dependent on SopB. To investigate whether ubiquitination is involved in this process, we compared the ability of wild-type SopB and SopB-K^6^R to induce Akt phosphorylation. HeLa cells were infected with Δ*sopB-sigE* bacteria, complemented with either pWSKDE-GSK or pWSKDE-K^6^R-GSK. At various times p.i., cells were lysed and subject to immunoblotting with antibodies against phospho-GSK (translocated SopB), GSK tag (total SopB), phospho-Ser473-Akt (activated Akt) and Akt pan (total Akt) (Fig. 7A). Like for wild-type SopB, translocated SopB-K^6^R accumulated with time in epithelial cells, although only the 60 kDa, unmodified, species was detected. The total amount of SopB-K^6^R displayed a similar trend with time. The Akt activation kinetics induced by the two bacterial strains were similar, with a peak of Ser473-phospho-Akt at 1 h p.i. However, infection with bacteria expressing the SopB-K^6^R mutant caused a threefold to fivefold increase in the peak intensity of phospho-Akt signal, as well as sustained Akt phosphorylation up to 4 h p.i.

Akt activation is a multistep process that involves recruitment from the cytosol to the plasma membrane where phosphorylation on Ser473 and Thr308 residues is followed by release of the now active kinase into the cytosol where it can target a variety of substrates (Franke, 2008). Recently, it has also been shown that Akt can also be activated on a subset of endosomes (Schenck *et al*., 2008). As SopB is an inositol phosphatase that affects the concentration of membrane phospholipids, we next used confocal microscopy to assess whether SopB-K^6^R affects the levels of phosphorylated Akt on membranes. HeLa cells expressing EGFP-mAkt1 were infected with Δ*sopB-sigE* bacteria, or Δ*sopB-sigE* bacteria complemented with pWSKDE or pWSKDE-K^6^R. The Δ*sopB-sigE* mutant was used as a negative control because it does not induce Akt phosphorylation (Steele-Mortimer *et al*., 2000). At various times up to 90 min p.i., cells were fixed and processed for immunofluorescence staining. Before permeabilization, cells were incubated with fluorescently conjugated wheat germ agglutinin (WGA) to label the plasma membrane. Subsequently, monolayers were permeabilized and immunostained using anti-phospho-Ser473-Akt antibodies. Plasma membrane ruffles, which are induced by *Salmonella* at the site of invasion and persist after bacterial internalization (Kubori and Galan, 2003), were identified using spinning disc confocal microscopy and single optical sections were acquired in the far-red (WGA, plasma membrane), green (EGFP-Akt) and red (phospho-Akt) channels. Subsequently the ruffle was selected by thresholding on the WGA-Alexa Fluor® 647 channel. The ratio of the average pixel intensity for the phospho-Ser473-Akt signal to EGFP-Akt fluorescence (R_*pAkt/Akt*_) was subsequently determined for each ruffle (Fig. 7B). In this microscopy-based assay, R_*pAkt/Akt*_ for Δ*sopB-sigE* bacteria was < 1 at all time points. In contrast, infection with bacteria complemented with plasmid-borne SopB resulted in Akt phosphorylation at the plasma membrane, as indicated by R_*pAkt/Akt*_ values > 1. Infection with bacteria expressing SopB-K^6^R also caused the accumulation of phospho-Akt in plasma membrane ruffles, at levels comparable to wild-type SopB at 30 min p.i., but at significantly greater levels at 60 and 90 min p.i. (Fig. 7B). These differences were not attributable to changes in the amount of EGFP-Akt recruited to plasma membrane ruffles (results not shown). Notably, we never detected EGFP-Akt or activated Akt on the SCV over the studied time-course (results now shown). This microscopy datum concurs with the immunoblot data from whole cell lysates (Fig. 7A) and together these experiments demonstrate that ubiquitination of SopB regulates its biological activity at the plasma membrane, where it mediates phosphorylation of Akt.

## Discussion

Here we have studied the contribution of ubiquitination to the *Salmonella* virulence factor, SopB, a type III effector that is essential for Akt activation at the site of bacterial entry into host epithelial cells. Using a combination of mass spectrometry and site-directed mutagenesis, we have shown that at least six of the 50 lysine residues in SopB are sites of ubiquitination, specifically mono-ubiquitination. Of the six modified lysine residues, five are clustered at the N-terminus of SopB and one is at the C-terminus. While a three-dimensional structure of SopB is not yet available, we predict that these residues are exposed on the surface of SopB, because ubiquitin is preferentially added to lysines present in surface-exposed loops (Catic *et al*., 2004). Recently, two other groups have also reported ubiquitin conjugation sites of SopB. Rogers *et al*. identified two of the same residues (Lys19 and Lys541), although this was not confirmed by mutagenesis (Rogers *et al*., 2008). Patel *et al*. identified two ubiquitinated tryptic peptides of SopB by mass spectrometry and subsequently mutated nine lysines at the amino terminus of SopB to demonstrate that all of these residues can be modified. Individual substitution of the identified lysine residues had little effect on SopB ubiquitination and mutation of all identified residues reduced, but did not eliminate, ubiquitination (this manuscript and Patel *et al*., 2009). Such redundancy of ubiquitin attachment sites is well documented for numerous mammalian proteins (Hou *et al*., 1994; Marotti *et al*., 2002; Lee *et al*., 2004; Huang *et al*., 2006; Meray and Lansbury, 2007). Furthermore, mutation of target lysine residues sometimes can lead to the modification of lysine residues that do not normally participate as ubiquitination sites (Hou *et al*., 1994; Marotti *et al*., 2002). This promiscuity of ubiquitin conjugation sites and the fact that only a fraction of the protein is modified define some of the challenges faced in addressing the biochemical aspects of protein ubiquitination.

*Salmonella enterica* serovars differ greatly in their host specificity and the type of disease they cause. Some of these serovars are highly host adapted, including Gallinarum, which only infects birds, and Typhi, only higher primates, whereas others such as Typhimurium have a broad host range. Likewise, the repertoire of type III effectors is not the same for all these serovars. For example, effectors encoded by phages or phage remnants, including AvrA, SopE, SspH1 and SseI, are absent from many serovars (Tsolis *et al*., 1999; Prager *et al*., 2000; Hansen-Wester *et al*., 2002; Rahman *et al*., 2005). By contrast, SopB appears to be prevalent (Prager *et al*., 2000; Mirold *et al*., 2001; Streckel *et al*., 2004; Rahman, 2006). The deduced amino acid sequence for full-length SopB is available for the following *S. enterica* serovars in GenBank; serovar Agona (GenBank accession number YP_002145960), serovar Choleraesuis (AAC33723), serovar Dublin (AAC33723), serovar Enteriditis (CAR32537), serovar Gallinarum (CAR36869), serovar Hadar (EDZ33941), serovar Heidelberg (YP_002045092), serovar Javiana (EDZ05526), serovar Kentucky (EDZ19176), serovar Newport (YP_002040347), serovar Saintpaul (EDZ10612), serovar Schwarzengrund (YP_002114089), serovar Typhi (NP_455588), serovar Virchow (ZP_03216329) and serovar Weltevreden (EDZ28428). Interestingly, all six identified ubiquitin conjugation sites (Lys13, Lys19, Lys23, Lys37, Lys41 and Lys541) are conserved in these SopB sequences. Such conservation implies that SopB and its ubiquitination are required for pathogenesis irrespective of host or serovar differences. Two of these lysine residues, Lys19 and Lys41, are also conserved in the SopB homologue from *Shigella*, IpgD (Allaoui *et al*., 1993; Hong and Miller, 1998), although whether IpgD is ubiquitinated remains to be determined.

SopB is an inositol phosphatase that can hydrolyse a number of inositol phosphates and phosphoinositides *in vitro* (Marcus *et al*., 2001). Interestingly, ubiquitination is known to regulate the activity of some mammalian inositol phosphatases. For example, the E3 ubiquitin ligase NEDD4-1, poly-ubiquitinates PTEN (phosphatase and tensin homologue deleted on chromosome 10), an inositol 3′-phosphatase that is a central negative regulator of phosphatidylinositol 3-kinase (PI3K) signalling, leading to its proteasomal degradation and thereby limiting its actions on phosphatidylinositol (3,4,5)-trisphosphate at the plasma membrane (Wang *et al*., 2007). NEDD4-1 also mono-ubiquitinates PTEN, inducing its translocation to the nucleus (Trotman *et al*., 2007). Therefore, ubiquitination of PTEN controls both its cellular localization and stability. Another inositol 3′-phosphatase, myotubularin-related protein 4, localizes to endosomes and is also poly-ubiquitinated by NEDD4, although the role of this post-translational modification is unknown (Plant *et al*., 2009). It might prove that ubiquitin-mediated control of inositol phosphatase function is more widespread than currently appreciated.

Upon bacterial contact with host cells, SopB is delivered to the cytoplasmic surface of the plasma membrane where it participates in plasma membrane ruffling and signalling events, and after bacterial entry it continues to be translocated for many hours, localizing to the SCV. Here we have shown that one role of SopB ubiquitination is to downregulate its activity at the plasma membrane. This concurs with the recent results of others (Patel *et al*., 2009). While this might be an indirect effect attributable to mutagenesis-induced changes in SopB enzymatic activity, it seems much more likely that it is directly due to ubiquitin-dependent changes in localization. Patel *et al*. (2009) have shown using an invasion-defective, translocation-competent *Salmonella* strain, that while SopB is internalized from the plasma membrane into endocytic compartments, ubiquitin-defective SopB is retained at the plasma membrane. Ubiquitin, primarily mono-ubiquitin, is well known to function as an internalization signal for eukaryotic plasma membrane proteins (Mukhopadhyay and Riezman, 2007), but this is the first example for a bacterial virulence protein. Ubiquitination of some plasma membrane receptors controls the amplitude and kinetics of signalling resulting from their engagement (Marmor and Yarden, 2004). Upon growth factor stimulation, these transmembrane proteins are mono-ubiquitinated, driving their internalization and sorting them to the endosomal/lysosomal pathway (Haglund *et al*., 2003; Staub and Rotin, 2006). While ubiquitin-deficient plasma membrane receptors are internalized normally, their turnover by lysosomal degradation is delayed, but not prevented (Huang *et al*., 2006). This leads to increased and prolonged PI3K/Akt signalling in the case of granulocyte colony-stimulating factor receptor (Ai *et al*., 2008). Conversely, overexpression of the E3 ubiquitin ligase cbl-b enhances ubiquitination of the epidermal growth factor receptor, shortening the duration of its signalling (Ettenberg *et al*., 1999). It is important to note that ubiquitination of plasma membrane receptors does not lead to an all-or-nothing effect, but rather modulates the intensity and duration of signalling events, serving a ‘fine-tuning’ role. Ubiquitin-mediated downregulation of signalling is not limited to transmembrane receptors at the plasma membrane because we, and others (Patel *et al*., 2009), observe a similar phenotype for SopB-dependent signalling.

While SopB is translocated by T3SS1 immediately upon contact with host cells, we did not detect modification of translocated SopB until ≥ 1 h p.i. The onset of SopB ubiquitination is coincident with the peak of Akt phosphorylation and leads to the efficient downmodulation of Akt activation at the plasma membrane. By contrast, we found no evidence for SopB ubiquitination being involved in SCV biogenesis. Unlike Akt activation, Rab5 and LAMP1 recruitment to the SCV and PI(3)P formation are affected, but not absolutely dependent, on SopB. SCV acquisition defects are only observed ≤ 40 min p.i. for a *sopB* deletion mutant (this manuscript and Mallo *et al*., 2008), and thereafter SCV maturation is indistinguishable from wild-type bacteria. The lack of influence of SopB ubiquitination on SCV trafficking is seemingly in agreement with the time frame of SopB-dependence (≤ 40 min p.i.) and kinetics of SopB modification (≥ 1 h p.i.). However, others have reported that *Salmonella* expressing ubiquitin-deficient SopB are defective for Rab5 recruitment to the SCV at 10–20 min p.i. SopB phosphatase activity is required for the presence of active Rab5 on the SCV, which recruits the PI3K Vps34, leading to PI(3)P production on the SCV (Mallo *et al*., 2008). It is difficult to reconcile how Rab5 recruitment can be affected by SopB ubiquitination (Patel *et al*., 2009), but not PI(3)P production (this study) and further studies are required to resolve this discrepancy.

Ubiquitination can alter protein location (Schnell and Hicke, 2003). While SopB remains peripherally bound to membranes via hydrophobic interactions regardless of its ubiquitination status (this report and Patel *et al*., 2009), we have shown that ubiquitination is required for the persistence of SopB on the SCV. Analogous to this, the association of many yeast and mammalian proteins with endosomes and their subsequent sorting by the endocytic machinery is largely controlled by their mono-ubiquitination (Raiborg *et al*., 2003). With less SopB being present on the SCV, this might explain the intracellular replication defect of bacteria expressing ubiquitin-defective SopB (Patel *et al*., 2009). However, another late event know to be affected by SopB, SCV positioning (Wasylnka *et al*., 2008), does not appear to depend upon ubiquitination (our unpublished results). Given that translocated SopB persists for many hours on the SCV, we believe that additional SopB-dependent functions will be discovered. Identification of the E3 ubiquitin ligase that targets SopB and protein binding partners of ubiquitinated SopB are key to clarifying the contribution of ubiquitination to activity and/or localization of this *Salmonella* effector.

## Experimental procedures

### Bacterial strains and plasmids

The wild-type strain *S. enterica* serovar Typhimurium SL1344 (Hoiseth and Stocker, 1981) and the Δ*sopB-sigE* mutant (Knodler *et al*., 2006) were as previously described.

To construct the complementing plasmid, pWSKDE (see Table S1 for a list of plasmids used in this study), the *sopB-sigE* open reading frames and approximately 400 bp of upstream region was amplified from pACDE (Marcus *et al*., 2001) with the oligonucleotides SigD-Hind-F and SigD-Nhe-R with the Expand High Fidelity PCR System (Roche) (see Table S2 for all oligonucleotide sequences used in this study). The resulting amplicon was digested with HindIII/NheI and ligated into HindIII/XbaI-digested pWSK29 (Wang and Kushner, 1991). The pWSKDE-K^6^R mutant (K13R K19R K23R K37R K41R K541R mutations) was generated by site-directed mutagenesis with the Quikchange kit (Stratagene).

To monitor the translocation of SopB by immunoblotting, a 13-residue tag derived from human GSK was fused to the C-terminus of SopB by overlap extension PCR. *sopB* and approximately 400 bp of upstream region was amplified from pWSKDE with the oligonucleotides SigD-Hind-F and SigD-GSK-R. The *sopB-sigE* intergenic region and *sigE* were amplified from pWSKDE with the oligonucleotides SigD-GSK-F and SigD-Nhe-R. After a second round of PCR with a mix of these two amplicons and the oligonucleotides SigD-Hind-F and SigD-Nhe-R, the product was digested with HindIII/NheI and ligated into HindIII/XbaI-digested pWSK29 to create pWSKDE-GSK. Similarly, pWSKDE-K^6^R was used as a template for amplification to create pWSKDE-K^6^R-GSK.

To detect translocated SopB by immunofluorescence, tandem HA tags were fused to the C-terminus of SopB by overlap extension PCR. pWSKDE or pWSKDE-K^6^R served as templates with the oligonucleotide pairs SigD-Hind-F and SigD-2HA-R and SigD-HA-F and SigD-Nhe-R for the initial PCR amplifications. Secondary PCR reactions and cloning were as described above, generating pWSKDE-2HA and pWSKDE-K^6^R-2HA.

For ectopic expression in mammalian cells, *sopB* was amplified from *S*. Typhimurium SL1344 genomic DNA with the oligonucleotides Myc-SigD-F and pGAD-SigD-R. The amplicon was digested with EcoRI and BglII and ligated into the corresponding sites of pCMV-Myc (Clontech) to create pCMV-Myc-SopB. pCMV-Myc-SopB K^6^R was generated with the QuikChange site-directed mutagenesis kit (Stratagene).

For ectopic expression of EGFP-tagged Akt, the open reading frame of murine Akt1 was amplified from GFP-HA-Akt (Watton and Downward, 1999) with the oligonucleotides EGFP-mAkt1-F and EGFP-mAkt1-R. The amplicon was digested with BglII/SalI and ligated into the corresponding sites of pEGFP-C1 (Clontech) to create EGFP-mAkt1. The plasmid encoding for 2xFYVE-EGFP was a gift from Mark Jepson (Pattni *et al*., 2001).

Plasmids encoding for HA-tagged ubiquitin (Lim *et al*., 2005) and Ubl modifiers, ISG15 (Kim *et al*., 2004), NEDD8 (Kamitani *et al*., 1997) and SUMO1 (Kamitani *et al*., 1998) were purchased from Addgene (Addgene plasmids 17608, 12444, 18711 and 17359 respectively).

All plasmid sequences were verified by DNA sequencing.

### Generation of polyclonal SopB antibodies

Murine polyclonal antibodies against SopB were prepared as described (Lacy and Voss, 1986), with some modifications. Female BALB/c mice (Jackson Laboratory) were inoculated subcutaneously at two sites with 8 μg purified recombinant SopB (kindly provided by Dr Sandra Marcus) in 0.2 ml Ribi Adjuvant (GlaxoSmithKline). Mice were boosted after 2 weeks with another 8 μg SopB resuspended in Ribi Adjuvant. A second boost was prepared in Complete Freund's Adjuvant (Sigma-Aldrich) because Ribi Adjuvant was no longer commercially available. Mice were bled and titres checked by immunoblot blot using recombinant SopB. Mice were given two doses of 0.5 ml pristine by intraperitoneal injection 1 week apart followed 1 week later by 5 × 10^6^ SP2 cells i.p. in 0.5 ml Hank's balanced salt solution (HBSS, Mediatech). Mice developed ascites 3 weeks later and ascites fluid was collected over 3 days, pooled and stored at −20°C. This immunization protocol was approved by the Rocky Mountain Laboratories Animal Care and Use Committee.

### Bacterial infection of mammalian cells

HeLa adenocarcinoma epithelial cells (ATCC CCL-2) were grown in Eagle's modified medium (Mediatech) containing 10% (v/v) heat-inactivated fetal calf serum (Invitrogen) at 37°C with 5% CO_2_. Cells seeded in 6 well plates, or on glass coverslips in 24 well plates, were infected 18–24 h after seeding with *S*. Typhimurium grown under invasion optimizing conditions (Knodler *et al*., 2006). Cells were serum-starved for 3 h prior to infection and maintained in serum-free media for the entire infection, unless otherwise indicated. Bacteria were added to HeLa cells at a MOI of ∼50 and invasion allowed to proceed at 37°C for 10 min. Monolayers were washed three times in HBSS, chased for 20 min in antibiotic-free serum-free media and then incubated in serum-free growth media containing 50 μg ml^−1^ gentamicin for 1 h, followed by 10 μg ml^−1^ gentamicin for the remainder of the experiment.

### Inhibitor experiments

To stop bacterial protein synthesis after bacterial internalization, 10 μg ml^−1^ tetracycline (Sigma) was added to the growth medium. This concentration was determined to be bacteriostatic (not shown). For Fig. 1B, tetracycline was added at 30 min p.i. For SopB stability experiments (Fig. 3B), tetracycline was added at 2 h p.i. To prevent bacterial transcription (Fig. 1C), 5 μg ml^−1^ rifampicin (Sigma) was added to the growth medium after the initial 10 min infection and maintained throughout.

### Taqman® real-time PCR analysis

HeLa cells were seeded in 6 well plates the day prior to infection (2 × 10^5^ cells/well) and infected with 5 μl invasive *Salmonella* (MOI ∼50) as described above. Bacterial and mammalian RNA and DNA were extracted from infected monolayers using TRIzol^®^ (Invitrogen) as described previously (Drecktrah *et al*., 2006). Briefly, DNA was removed from the RNA fraction by treating with DNase I (Invitrogen) according to the manufacturer's instructions. cDNA was synthesized using 2 μg RNA and the Taqman^®^ reverse transcription reagent kit (Applied Biosystems) according to the manufacturer's instructions. Primers and probes were designed using Primer Express^®^ version 1.5 (Applied Biosystems). Real-time PCR was performed in 384 well plates using a 7900 HT Sequence Detection System (Applied Biosystems) with primers and probes for *sopB* and *ssaH* (Drecktrah *et al*., 2006) and *ftsZ*. The amount of *sopB* and *ssaH* cDNA was normalized to *ftsZ* cDNA for each time point and condition.

### Subcellular fractionation and membrane extractions

Fractionation of infected host cells was as described previously (Knodler *et al*., 2003), with the following modifications. Four 10 cm dishes of HeLa cells were infected with 50 μl invasive *Salmonella* as described above. At 1.5 h p.i., cells were collected by scraping and then mechanically disrupted by passing through a 22-gauge needle. To one half of the sample, 5 ml cold homogenization buffer, containing protease inhibitor cocktail set III and phosphatase inhibitor cocktail set II (EMD Biosciences), was added. Nuclei were pelleted by centrifugation at 1000 *g* for 5 min at 4°C (P1K). The supernatant was harvested and centrifuged again at 6000 *g*, 4°C, to pellet bacteria (P6K). Both pellets were resuspended in 300 μl boiling 1.5× SDS-PAGE buffer. The other half was centrifuged at 6000 *g* for 10 min at 4°C, the supernatant collected and ultracentrifuged at 100 000 *g* for 30 min at 4°C to separate membranes (P100K) from cytosol (S100K). Boiling 6× SDS-PAGE sample buffer was added to the supernatant and the pellet was resuspended in 300 μl boiling 1.5× SDS-PAGE buffer. Equal volumes of each fraction were loaded onto SDS-PAGE gels and subject to immunoblotting.

For membrane extraction studies, HeLa cells in 10 cm dishes were infected with *Salmonella* as above. At 1.5 h p.i., cells were mechanically disrupted in 300 μl cold homogenization buffer containing protease inhibitor cocktail set III and phosphatase inhibitor cocktail set II (EMD Biosciences) and centrifuged at 6000 *g* for 10 min at 4°C. The supernatant was collected and divided into four aliquots. Samples were ultracentrifuged at 50 000 *g* for 30 min at 4°C to pellet host cell membranes. Membranes were resuspended in 150 μl phosphate-buffered saline (PBS), 1 M NaCl, 0.1 M Na_2_CO_3_ pH 11.5 or 1% TX-100. After 30 min incubation on ice, samples were ultracentrifuged at 100 000 *g*, 4°C, for 30 min. Boiling 6× SDS-PAGE sample buffer (50 μl) was added to supernatants. Membranes were resuspended in 150 μl each respective extraction buffer and 50 μl boiling 6× SDS-PAGE sample buffer added. Equal volumes were loaded onto SDS-PAGE gels and subject to immunoblotting.

### Transient transfection of mammalian cells

HeLa cells were seeded in 10 cm dishes, 6 well plates or on glass coverslips in 24 well tissue culture plate(s) 6–8 h prior to transfection. Plasmid DNA was prepared using the Perfectprep Plasmid Midi kit (Eppendorf) according to the manufacturer's instructions and cells transfected with FuGENE® 6 reagent (Roche). Cells were lysed 18–20 h post transfection in 150 μl boiling 1.5× SDS-PAGE sample buffer for immunoblotting (6 well plates) or processed for immunoprecipitation (10 cm dishes) or immunofluorescence (glass coverslips in 24 well plates) as described below.

### Immunoprecipitation and mass spectrometry

Twelve 10 cm dishes of HeLa cells were transfected with pCMV-Myc-SopB as described above and incubated for 18 h in Eagle's modified medium containing fetal calf serum. Monolayers were then washed twice in ice-cold PBS and collected by scraping into PBS. After centrifugation at 1000 *g* for 10 min at 4°C, cells were lysed on ice for 20 min in 1.8 ml of 50 mM Tris-HCl pH 8.0, 150 mM NaCl, 0.5% TX-100, 1 mM EDTA containing protease inhibitor cocktail set III and phosphatase inhibitor cocktail set II (EMD Biosciences). Lysates were clarified by centrifugation at 5000 *g* for 15 min at 4°C. The supernatant was pre-cleared by incubation with 35 μl Protein A Sepharose (Amersham) for 1 h at 4°C and then incubated for 2 h with 10 μl anti-Myc antibody (9B11; Cell Signalling Technology). Immune complexes were collected with 35 μl Protein A Sepharose for 2 h at 4°C. Sepharose beads were washed four times in lysis buffer and once in lysis buffer without TX-100, and bound proteins were eluted by boiling in 1.5× SDS-PAGE sample buffer for 5 min. Proteins were separated by SDS-PAGE on 10% polyacrylamide gels (Bio-Rad) and visualized by GelCode Blue staining (Pierce). Analysis of ubiquitin conjugation sites was carried out at the Taplin Mass Spectrometry Facility, Harvard Medical School.

For co-immunoprecipitation experiments, three 10 cm dishes were transfected with plasmids encoding HA-tagged ubiquitin or HA-tagged Ubl modifiers and infected 18–20 h later with *Salmonella* at a moi ∼150 for 10 min. Cells were not serum-starved in these experiments. At 2 h p.i., cells were collected by scraping into cold PBS and lysed on ice for 15 min in 20 mM Tris-HCl pH 8.0, 150 mM NaCl, 1% TX-100, 1 mM EDTA containing protease inhibitor cocktail set III and phosphatase inhibitor cocktail set II (EMD Biosciences). Samples were centrifuged at 6000 *g* for 10 min, the supernatant collected and SopB immunoprecipitated with 1 μg rabbit polyclonal anti-SopB antibodies as described previously (Marcus *et al*., 2002).

### Immunofluorescence staining

HeLa cells seeded on glass coverslips were fixed in 2.5% (w/v) paraformaldehyde (PFA) for 10 min at 37°C, then permeabilized with 0.1% (w/v) saponin in 10% normal goat serum (NGS, Invitrogen) (NGS-saponin) in PBS or 10% normal donkey serum (Millipore) in PBS for 20 min at room temperature and immunostained as described (Knodler *et al*., 2003). Primary antibodies were: rabbit polyclonal anti-*Salmonella* LPS (1:2000 dilution, Difco) and mouse monoclonal antihuman LAMP1 (H4A3, 1:1500 dilution, Developmental Studies Hybridoma Bank) for LAMP1 recruitment analysis; or goat polyclonal anti-*Salmonella* (CSA-1, 1:1000 dilution, KPL) and mouse monoclonal anti HA.11 (clone 16B12, 1:1000 dilution, Covance) for immunolocalization of SopB-2HA. For 2xFYVE-EGFP recruitment to the SCV, extracellular and intracellular bacteria were distinguished by immunostaining as described previously (Celli *et al*., 2001). Extracellular bacteria were immunostained with mouse anti-*Salmonella* LPS antibodies (1:5000, clone 1E6, Biodesign International) prior to permeabilization and all bacteria were stained with rabbit anti-*Salmonella* LPS antibodies (1:2000, Difco) postpermeabilization. Only intracellular bacteria were scored for 2xFYVE colocalization. All Alexa Fluor^®^ secondary antibodies were from Invitrogen and used at 1:800 dilution. Samples were observed on a Nikon Eclipse E800 or TE2000 epifluorescence microscope equipped with a Plan Apo 60×/1.4 objective for quantitative analysis.

Confocal images were captured on a Carl Zeiss LSM 510 Meta confocal laser-scanning microscope. Confocal sections of 0.3 μm and image sizes of 1024 × 1024 pixels (for SopB-2HA, Fig. 5C) or 512 × 512 pixels (for EGFP-mAkt recruitment, Fig. 7C) were acquired. Image analysis and maximum intensity projections were performed with ImageJ v.1.4.1 (written by Wayne Rasband at the US National Institutes of Health and available at http://rsbweb.nih.gov/ij/download.html) and figures assembled using Adobe Photoshop CS2.

### Analysis of Akt recruitment to membrane ruffles

For recruitment of Akt to *Salmonella*-induced membrane ruffles, cells seeded on glass coverslips were transfected overnight with EGFP-mAkt1, infected with *Salmonella* and at the indicated times, monolayers were fixed in PFA as described above. Monolayers were incubated with 1 mg ml^−1^ Alexa Fluor^®^647-conjugated WGA (Invitrogen) in PBS for 5 min to label the plasma membrane, fixed for 5 min in 2.5% w/v PFA at room temperature, washed, permeabilized with NGS-saponin and immunostained with mouse monoclonal anti-Ser473-phospho-Akt antibodies (1:200 dilution, 587F11, Cell Signaling Technology) followed by Alexa Fluor^®^568-labelled goat anti-mouse IgG (Invitrogen). Confocal images were acquired on an Ti-E inverted microscope with a 60 × 1.4 NA objective (Nikon Instruments), fitted with a Nipkow-spinning disk (Perkin-Elmer), a custom solid state laser launch with 488 nm, 561 nm and 643 nm laser lines (Prairie Technologies), emission filters of 525/50, 600/45 and 700/75 nm (Chroma Technologies) in a high speed filter wheel controlled by a Sutter 10-2 filter wheel controller (Sutter Instruments), a Cascade II:512 EM-CCD camera (Photometrics) and controlled by Metamorph 7.6.0 (Molecular Devices). Laser light intensities and capture settings were kept constant throughout the experiment. Only cells with low EGFP expression levels were selected for analysis. Plasma membrane ruffles were selected from a plane above the main cell body by thresholding on the WGA channel. The resulting region of interest was transferred to the EGFP-Akt and phospho-Ser473-Akt images and the ratio of mean fluorescent intensity for phospho-Ser473-Akt : EGFP-Akt in the membrane ruffle determined.

### Immunoblotting

Proteins were separated by SDS-PAGE, transferred to nitrocellulose (Bio-Rad) and blocked in Tris-buffered saline containing 5% non-fat dried milk powder (TBST-milk) or 5% bovine serum albumin (TBST-BSA) for 1 h at room temperature. Blots were incubated overnight at 4°C with the following primary antibodies: rabbit anti-phospho-GSK-3β Ser9 (Cell Signaling Technology) 1:2000 dilution in TBST-BSA, rabbit anti-GSK-3β-tag (Cell Signaling Technology) 1:5000 dilution in TBST-BSA, rabbit monoclonal anti-phospho-Ser473-Akt (193H12, Cell Signaling Technology) 1:10 000 dilution in TBST-BSA, rabbit monoclonal anti-Akt pan (11E7, Cell Signaling Technology) 1:2000 in TBST-BSA, mouse monoclonal anti-HA.11 purified (16B12, Covance) 1:20 000 in TBST-milk, mouse polyclonal anti-SopB 1:2000 in TBST-milk, rabbit anti-calnexin (Stressgen) 1:40 000 in TBST-milk, rabbit anti-calreticulin (Affinity Bioreagents) 1:10 000 in TBST-milk, rabbit anti-lamin A/C (Cell Signaling Technology) 1:5000 in TBST-milk, mouse anti-Hsp27 (G31, Cell Signaling Technology) 1:10 000 in TBST-milk, mouse anti-LAMP1 (H4A3, Developmental Studies Hybridoma Bank) 1:5000 in TBST-milk, mouse anti-DnaK (8E2/2, Stressgen) 1:40 000 in TBST-milk. Immunoblotting with rabbit polyclonal anti-SopB antibodies was as described previously (Knodler *et al*., 2006). Blots were washed in TBST or TBST-milk, incubated with affinity purified HRP-conjugated goat anti-rabbit or horse anti-mouse IgG (Cell Signaling Technology) 1:20 000 in TBST-milk for 1 h, washed and developed using enhanced chemiluminescence according to the manufacturer's directions (SuperSignal West Femto Maximum Sensitivity substrate, Thermo Scientific).

## Supporting information

**Table S1.** Plasmids used in this study.

**Table S2.** Oligonucleotides and probes used in this study.
