# Supplementary material for: Ubiquitination of the bacterial inositol phosphatase, SopB, regulates its biological activity at the plasma membrane
Source: Cell Microbiol. 2009 Aug 4;11(11):1652–70. doi: 10.1111/j.1462-5822.2009.01356.x (PMC2762020; doi:10.1111/j.1462-5822.2009.01356.x)
Supplement: Supplementary file 1 [file cmi0011-1652-SD1.doc]

**Supplementary Table 1**

Plasmids used in this study

| Plasmid name | Description | Source or Reference |
| --- | --- | --- |
| pWSK29 | Low copy bacterial expression vector, Amp^R^ | (Wa*ng et a*l., 1991) |
| pWSKDE | SopB and SigE in pWSK29 | This study |
| pWSKDE-K^6^R | SopB(K13R K19R K23R K37R K41R K541R) and SigE in pWSK29 | This study |
| pWSKDE-GSK | SopB-GSK and SigE in pWSK29 | This study |
| pWSKDE-K^6^R-GSK | SopB(K13R K19R K23R K37R K41R K541R)-GSK and SigE in pWSK29 | This study |
| pWSKDE-2HA | SopB-2HA and SigE in pWSK29 | This study |
| pWSKDE-K^6^R-2HA | SopB(K13R K19R K23R K37R K41R K541R)-2HA and SigE in pWSK29 | This study |
| pCMV-Myc | N-terminal c-Myc epitope tag mammalian expression vector | Clontech |
| pCMV-Myc-SopB | Myc-tagged SopB in pCMV-Myc | This study |
| pCMV-Myc-SopB K^6^R | Myc-tagged SopB(K13R K19R K23R K37R K41R K541R) in pCMV-Myc | This study |
| pRK5-HA-Ubiquitin-WT | HA-tagged ubiquitin | (L*im et a*l., 2005) |
| pCAGGS-5HA-mISG15 | HA-tagged murine ISG15 | (K*im et a*l., 2004) |
| HA-NEDD8 | HA-tagged NEDD8 | (Kamita*ni et a*l., 1997) |
| SRa-HA-SUMO1 | HA-tagged SUMO1 | (Kamita*ni et a*l., 1998) |
| pEGFP-C1 | N-terminal EGFP tag fusion mammalian expression vector, Kan^R^ | Clontech |
| EGFP-mAkt1 | Murine Akt1 in pEGFP-C1 | This study |
| 2xFYVE-EGFP | Tandem FYVE finger of Hrs fused to EGFP | (Patt*ni et a*l., 2001) |

**Supplementary Table 2**

Oligonucleotides and probes used in this study

| Name | Sequence (5’ to 3’) |
| --- | --- |
| SigD-Hind-F | CCC AAG CTT TCT GTT CAA GCA TGG AAT |
| SigD-Nhe-R | CTA GCT AGC TCA TTA AGA AAG TAT GTT GAC |
| SigD-GSK-F | ATG AGC GGC CGC CCG CGC ACC ACC AGC TTT GCG GAA AGC TGA GTC TTG AGG TAA CTA TAT |
| SigD-GSK-R | GCT TTC CGC AAA GCT GGT GGT GCG CGG GCG GCC GCT CAT AGA TGT GAT TAA TGA AGA AAT |
| SigD-2HA-R | CGC ATA ATC CGG CAC ATC ATA CGG ATA CGC ATA ATC CGG CAC ATC ATA CGG ATA AGA TGT GAT TAA TGA AGA AAT |
| SigD-2HA-F | TAT CCG TAT GAT GTG CCG GAT TAT GCG TAT CCG TAT GAT GTG CCG GAT TAT GCG TGA GTC TTG AGG TAA CTA TAT G |
| Myc-SigD -F | CCG GAA TTC AAA TAC AGA GCT TCT ATC AC |
| pGAD-SigD-R | GGA AGA TCT TCA AGA TGT GAT TAA TGA AGA |
| EGFP-mAkt1-F | Gga aga tct atg aac gac gta gcc att gtg |
| EGFP-mAkt1-R | A cgc gtc gac tca ggc tgt gcc act ggc tga |
| ftsZ-forward | TCG TTA TCA GCA GCA CGG TAT G |
| ftsZ-reverse | ACC ACT TTC GCG ACC GTT T |
| ftsZ-probe | (VIC™)CGC CGC TGA CGC AGG AGC A(TAMRA) |

Engineered restriction sites are underlined.
